# Understanding the psychological impact of identifying carrier status on young adults: A qualitative study exploring peer reactions

**DOI:** 10.1002/jgc4.1903

**Published:** 2024-04-26

**Authors:** Edie Bowen, John Langston, Harriet Fletcher, Julia Domek, Fiona Ulph

**Affiliations:** ^1^ Manchester Centre for Health Psychology, Division of Psychology & Mental Health, School of Health Sciences, Faculty of Biology, Medicine and Health University of Manchester, Manchester Academic Health Science Centre Manchester UK

**Keywords:** carrier testing, communication, COVID‐19, newborn screening, qualitative methods, stigma

## Abstract

The benefits and harms of identifying carriers in childhood have long been debated with European Guidelines advising against this practice. Yet over a thousand carriers are identified via newborn bloodspot screening per year in the United Kingdom alone. One of the concerns about identification is the impact it has on an individual's identity. This, in part, will be determined by how parents and peers view carriers, particularly during young adulthood. To address the paucity of research looking at how carriers are perceived by peers, this study sought to explore the views of young adults, who themselves are not carriers, toward carriers. As the narratives around COVID‐19 increased, the salience of the term “carrier”, the impact of such narratives on perceptions, was also explored. Twenty‐five 18–25 year olds participated in a diary‐interview study in the United Kingdom during 2021 to explore their perceptions of carriers via hypothetical scenarios. Data were analyzed using thematic analysis. Interviewees believed carriers would experience stigma—including societal and self‐stigma. This was because people used existing illness beliefs to make sense of carrier status about which they had low levels of understanding. Interviewees believed carriers would experience challenges in familial and romantic relationships due to others' judgments. They also believed parents of carriers would experience a burden around making reproductive decisions, with clear views on what society would view as acceptable choices. Importantly interviewees felt knowledge of ones' own carrier status conferred complex communication challenges within relationships. These findings suggest an urgent need for more research and support for young adults entering a key stage in life for identity formation who have knowledge of their carrier status. The results suggest that support targeted toward the carrier regarding navigating complex communication and targeted more broadly to avoid stigma based on misunderstanding should be researched and developed.

**What is known about this topic**

Research has previously shown that carrier information can lead to stigmatization of adults. European guidelines raise concerns about the impact of carrier identification in childhood but acknowledge there is a lack of research in this area.

**What this paper adds to this topic**

This is the first study to explore how young adults identified as autosomal carriers could be perceived by their peer group and the impact on their romantic relationships. Findings suggest that young adults expect carriers to disclose their status early in the formation of relationships, while simultaneously being concerned about the impact of disclosure on relationships.

## 1 INTRODUCTION

Newborn bloodspot screening (NBS) aims to identify babies with rare, serious, and treatable conditions. NBS also, in some countries, identifies carriers of autosomal recessive conditions such as cystic fibrosis and sickle cell. As the health implications of being a carrier are primarily reproductive (Hussein et al., 2018), such identification in childhood has been viewed as problematic (Borry et al., 2006; Royal College of Physicians et al., 2022). Debates exist around the benefits and harms of identifying carriers (Armstrong et al., 2020) and telling a child their carrier status (Miller et al., 2009). The piloting of whole‐genome sequence‐based NBS in the United Kingdom, which enables many more conditions (an expansion from 9 to 200+ conditions) to be identified and, to our understanding, will result in some families being told their child is a carrier following subsequent testing. This has intensified calls to better understand the benefits and harms of NBS results including carriers (Ulph & Bennett, 2022). When considering the benefits and harms of identifying carriers via NBS from a psychological perspective, we must consider this from the perspective of the parents in the immediate term and the child in the longer term.

### 1.1 Carrier identification—Impact on parents

The process of determining carrier status often involves recalling parents to hospital for further tests. As such parents can undergo a period of uncertainty during which they may worry their child has a serious life‐limiting condition, this has been linked to depression, anxiety, exhaustion, and isolation (e.g., Chudleigh et al., 2016; Lang et al., 2011; Moran et al., 2007; Salm et al., 2012; Ulph et al., 2015). Ulph et al. (2015) conducted semi‐structured interviews with 67 family members of 51 infants identified as carriers via NBS. Many valued knowing about their child's carrier results, but found the disclosure extremely distressing. A common finding of such research is that parents are often able to recall their experiences a number of years later with great lucidity. Known as “flashbulb” memories (Brown & Kulik, 1977), these memories are often formed about events that are unexpected and severely emotionally arousing. Ulph et al. (2015) attributed this distress to two processes. Firstly, parents being unaware their children may be identified as carriers via NBS, and therefore assuming their child had the condition when contacted about results. Secondly, the way in which health professionals communicated the screening results amplified concerns that their child had the condition. That parents feel unprepared for screening results is a common finding internationally (Chudleigh et al., 2016; Moran et al., 2007; Salm et al., 2012; Schwan et al., 2019; White et al., 2021) and across result types. Research suggests that both the carrier result itself and the way this information is disclosed to parents have both immediate (Moran et al., 2007; Ulph et al., 2015) and long‐lasting (Lewis et al., 2006) consequences. How prescreening communication practices could be changed to better engage and prepare parents for such screening has been researched (Ulph et al., 2017) with repeated calls being made to improve such communication (Kai et al., 2009; Tluczek et al., 2022; Ulph et al., 2017, 2020). Proposed benefits of identifying carriers via NBS include parents and children becoming aware of the future reproductive risks (Miller et al., 2009), although whether this information truly does influence reproductive decisions has been hard to discern (Hayeems et al., 2008). Although there is support for knowing this information (Hayeems et al., 2008), there are still many misunderstandings about the difference between being a carrier and having the condition, and adults have been shown to believe carriers have more health issues (Hayeems et al., 2008; Mayo‐Gamble et al., 2018). It is important to establish whether young adult peers will also do this.

### 1.2 Impact on young adults

Emerging adulthood (a pivotal life stage 18–25 years old; Arnett, 2000) is a critical time for identity formation as individuals explore love, work, and world views. Within this, peer relationships and the ability to form romantic relationships are crucial parts of identity formation (Joshi & Deuskar, 2016). Parents plan to disclose carrier status to their children in adolescence or young adulthood when the individual starts becoming sexually active (Ulph et al., 2014), so young adults identified as carriers via NBS should be aware of their status. Although guidelines about genetic testing in childhood emphasize the need to balance benefits and harms of carrier identification (Royal College of Physicians et al., 2022), there is minimal research into the experiences of knowing you are a carrier at this age. Research into school‐based carrier testing suggests that children do not have adverse reactions to learning such information (Barlow‐Stewart et al., 2003; Mitchell et al., 1993, 1996). However, what is not always clear is whether the children fully understood their results, or if this adaptation can only be achieved in students studying relevant subjects when all peers understand what it means to be a carrier. Research with genetic counselors suggests that ensuring children understand their carrier status can be challenging, with few resources available (Ulph et al., 2010). This is a critical gap as during this age span peer acceptance is an important driver of psychological well‐being.

### 1.3 Psychological impacts of identification

Research has shown the detrimental effects of misinterpretation of carrier status (Mischler et al., 1998) leading to stigmatization and discrimination (Lewis et al., 2010; Ulph et al., 2011) even within communities with high levels of awareness (Kai et al., 2009). Specifically, there is evidence that genetic carriers experience both self and social stigmatization (Lewis et al., 2010). Social stigma involves the labeling of differences as undesirable, resulting in discrimination (Bathje & Marston, 2014), and self‐stigma is when a person agrees with and internalizes the stereotypes of the group they perceive themselves to belong in (Corrigan et al., 2010). Stamatoyannopoulos (1974) found that when notified of their status, carriers of sickle cell would feel embarrassed, inferior, or anxious, suggesting a harmful internalization of this label, potentially leading to adverse effects on identity. Evers‐Kiebooms et al. (1994) evaluated the emotional reaction to obtaining a carrier result for both carriers and non‐carriers. A comparison between the two groups' responses on the Health Orientation Scale (HOS) revealed that carriers had substantially lower positive feelings regarding themselves, in contrast with non‐carriers, describing themselves as weaker, less happy, and worse. Both groups ascribed more negative feelings toward genetic carriers than non‐carriers, suggesting the presence of societal stigma in relation to carrier identification. This was later replicated by Gordon et al. (2003), who reported identical findings in a larger sample (353 respondents, 47 of whom had been identified as cystic fibrosis carriers) and had previously been shown in sickle cell carriers (Wooldridge & Murray Jr., 1988). These authors concluded this was due to a stigmatizing effect of carrier identification. Of concern, all these papers included adults who received genetic counseling, making it even more necessary to explore the understanding of young adult carriers identified via NBS and their peers, neither of whom are likely to receive information from specialist health professionals. One study in the field investigated how 10‐ to 17‐year‐old non‐carriers would respond to a peer who discloses their carrier status (Noke, 2015). Findings showed that children's views were stigmatizing, and carriers were seen as “different,” with beliefs that they would have lowered self‐concept and would not fit into their peer group. Thus, although carrier status results are seen as medically benign, they may not be psychosocially benign (Ulph & Bennett, 2022).

### 1.4 Impact of COVID‐19

The impact that large‐scale public health emergencies can have on people's knowledge, attitudes, and perceptions has been recognized (Adefisoye, 2021) and it is accepted that people's health knowledge will have been impacted by the magnitude of the COVID‐19 pandemic (WHO, 2020). Relevant to this work, the terms “asymptomatic” and “carrier” were frequently used during the pandemic to refer to individuals without COVID‐19 symptoms who could transmit the virus (WHO, 2020). Such people were a health threat and to be avoided. This contrasts with carriers of genetic conditions who do not pose any health threat (Kai et al., 2009). There is concern that the use of words “asymptomatic” and “carrier” has been associated with being contagious and harmful to others. As such, this information may be used to form opinions of genetic carriers when people have little knowledge. Noke and Ulph (2014) have shown that, even when unable to confidently name features of genetic conditions, people use their pre‐existing knowledge to form assumptions about condition features. Furthermore, these misconceptions about genetic conditions were often stated in front of peers with confidence, which could lead others to assimilate them as fact. Although there have been a number of studies that have looked at the influence of risk perceptions on beliefs about COVID‐19 and engagement in various behaviors (Cipolletta et al., 2022), fewer have looked at how living through the pandemic altered risk perceptions of other conditions.

This study used qualitative methodology to explore young adults' views of carriers of a genetic condition and the possible impact that COVID‐19 has had on these. These young adults were not themselves carriers, so as to better understand peer reactions, which could influence carrier identity formation. In addition, we explored the views toward carriers generally, rather than focusing on one particular condition, so as to ensure that the perceptions about the condition itself were not driving the views.

## 2 MATERIALS AND METHODS

### 2.1 Participants

A convenience sample of 25 young adults (23 = female, 2 = male), aged 18–25 years, was recruited via an advert on a study recruitment site for psychology students at a University in the North of England. The advert explained the study and that those who knew they were carriers of a genetic condition were not invited to participate. We excluded carriers to ensure that the sample represented more closely reactions of peers toward carriers and because we could not be sure that views were not distressing to carriers. Most participants described a lack of previous genetic knowledge, with few participants confident in their understanding and knowledge of genetics.

### 2.2 Instrumentation and procedure

This two‐phase study used diaries and interviews to explore participants' views.

#### 2.2.1 Phase 1—Diary study

Diaries were used as they allow creativity and flexibility in participants' responses (Alaszewski, 2006), enable short‐term daily reflections (McDonnell et al., 2017) and so the collection of participants' thoughts and feelings as and when they occur, driving depth of consideration of the research topic. However, this method of research is not without its own issues, such as compliance and willingness to complete the diaries in detail (Stone et al., 2002), which in turn can affect the quality of the data (Elliott, 1997). Therefore, diaries are often used in addition to other methods, such as interviews (McDonnell et al., 2017). Interested participants contacted the researchers who sent them the participant information sheet. Twenty‐four hours later, participants were emailed the first diary entry. Returning this signified consent to take part in the diary study, so upon return, a consent form to participate in the interview study was also sent. Participants were emailed a diary sheet daily over the course of 7 days which was designed for this study by the research team including young adults and an experienced qualitative researcher with expertise in this topic. The initial diary entry explored participants' pre‐conceptions of carriers. Each day, participants completed a number of tasks, considering their initial thoughts around carriers, and considering the hypothetical situation of how they would feel if they were informed that either they themselves or their newborn baby was a carrier of a genetic condition and whom they think they would tell if they received this information. Participants were also asked to describe the characteristics of two stick people, one labeled as “carrier” and the other “non‐carrier,” further encouraging them to consider their thoughts on carriers prior to the interview. Finally, participants were asked whether their responses had been influenced by anything they had been exposed to about COVID‐19 that day. On completion of each daily response, they password protected the document and emailed it back to the researchers. Researchers reviewed the responses before conducting the interview, taking detailed notes, and interweaving them into the interview schedule. In this way, the diary entries shaped the interviews to an extent and provided prompts and areas to explore further.

#### 2.2.2 Phase 2—Interview

On‐line semi‐structured interviews enable the collection of rich data on participants' thoughts, feelings, and beliefs without being restricted by the researchers' pre‐formulated ideas (Berg, 2009) increasing the opportunity for unanticipated insights. Online interviews are convenient, flexible, and cost‐effective (Hewson, 2008; Horrell et al., 2015) aspects that participants appreciate (Deakin & Wakefield, 2013) and a necessity as the data were collected during the COVID‐19 lockdown in 2021. The interview schedule, developed by the team, covered similar topics to the diary: views of carriers, a vignette that their baby was diagnosed as a carrier, and reflection on whether COVID‐19 coverage affected their views. While the schedule was used across interviews, the sequence and phrasing of questions were altered to explore in more detail participants' responses, both in the interview and their diaries, and adapt questions to the individual and probe (Kvale, 1996). The interviewer made use of the screen share function on Zoom to show participants their diary entries in order to enquire further about their responses. Each interview lasted approximately 1 h. After the interviews, participants were emailed a debrief sheet. Interviews were transcribed verbatim removing identifiable information to ensure anonymity.

### 2.3 Reflexivity

All authors had previously undertaken qualitative research and used thematic analysis to identify patterns and themes across data (Braun & Clarke, 2006). EB had undertaken a placement in perinatal mental health. Her pre‐existing knowledge of the perinatal period informed the development of interview questions. JD, JL, and HF could not rely on tacit perinatal knowledge so asked more follow‐up questions to clarify participants' meanings.

### 2.4 Data analysis

A pragmatic decision was made not to present the diary data due to journal word limits that could have created a superficial summary of both data sets. As such, data analysis focused on the interview data as the diary responses were captured and reflected within these data. Analysis was conducted by EB, JD, JL, and HF with supervision from FU—reviewing and advising on each of the six stages.

Reflexive thematic analysis (TA) is a widely used qualitative analytic method that involves searching for themes or patterns across an entire data set (Braun & Clarke, 2021). It is commonly used to provide accessible accounts of complex data. We took a critical realist stance, which holds that while there is a knowable reality out there, people's experiences and accounts of this are shaped by the social world including their language (Braun & Clarke, 2021). Themes were developed inductively using both the semantic and latent levels, centered around both explicit and implicit meaning (Fereday & Muir‐Cochrane, 2006).

Data analysis followed the six stages outlined by Braun and Clarke (2021). We have highlighted these stages in bold with the exception of the sixth which is writing up. Firstly, **familiarization** of the data occurred by “repeated reading,” actively searching for meaning across participants' accounts, and noting any initial thoughts in the margins of the interviews at the semantic (or explicit) level. After familiarization with the breadth and depth of the data, researchers worked on the transcripts of the interviews they conducted and **initial ideas for coding** were noted and returned in order to facilitate the formal coding process and organization of data into codes or “meaningful groups” (Tuckett, 2005). A schedule was set so that the team met after the coding of each interview to discuss their codes and resolve queries regarding the coding process. Once the data were coded, codes and quotes were collated into a Microsoft Excel document creating a compilation of the coding. At this stage, each analyst (of the four) selected areas of the data to focus on and **generated initial themes** for their areas (such as romantic relationships, disclosure, and impact on self). Following this, **themes were developed and reviewed**. Through this process, some themes were collapsed and others were re‐named as subthemes. Finally, the coded data were then actively re‐read alongside the consequent themes to **refine, define, and name final themes** to ensure they formed a coherent pattern, all the while considering the validity of the themes in relation to the entire dataset. The analysts created a clear and internally coherent account with an associated narrative both within each individual theme and across each theme in relation to the research question. For this paper, the separate analyses were reviewed and combined to enable an account of the entire dataset and broader research question.

In exchange for taking part, participants received course credits. The study was approved by the University of Manchester Research Ethics Committee.

## 3 RESULTS

Three themes were created. “Stigma” included both societal and self‐stigma. These beliefs appeared to be driven by people using existing illness belief models to make sense of carrier status about which they had low levels of understanding. This resulted in people believing carriers were ill or suffered in some way. Those who had these beliefs, followed through in their explanations in the second theme “Impact on relationships” where some interviewees believed carriers would experience challenges in familial and romantic relationships due to others' judgments. They also believed parents of carriers would experience a burden around making reproductive decisions, with clear views on what society would view as acceptable choices. However, data in this theme showed there was some counterbalance where people could appreciate that carriers did not have the condition. Most, however, felt that knowledge of ones' own carrier status conferred complex communication challenges within relationships that are explored in the final theme “Communication Challenges.”

### 3.1 Stigma: Societal stigma

Almost all participants believed that carriers would be stigmatized at a societal level.

they tend to like label them as ohhh that girl with that genetic condition. So, I feel like that's what you become known for, so errr I guess people would label you and put you into that kind of… that stigma I guess. (P3)

Despite being aware of the potential for discrimination, some participants showed stigmatizing views themselves. Commonly, participants believed that carriers would be most different in romantic relationships:

I don't want it to become a strain on my personal relationships. If being a carrier means me and my future partner can't have children or if my parents wish for grandchildren. (P16)

Other, more general stigma was also seen in some participants:

I was thinking about how if the baby has inherited disease how is this linked to reincarnation […] Is it a punishment for people who did bad things during previous lives? (P19)

This participant indicates that he/she see being a carrier as a negative trait for the reason that the baby could be punished by having the condition they carry, suggesting an element of blame as well as stigma. Probing revealed that this participant felt that if there were any “good genetic carriers,” which they explained to mean the ability to pass on beneficial traits rather than conditions, then it would not be a bad thing to be a carrier. However, oftentimes stigma was much more implicit, with participants believing carriers would not lead completely normal lives:

whereas a non‐carrier doesn't really have that worry and sees themselves as completely normal and healthy (P16)

This idea that carriers were less healthy than non‐carriers was a common justification for stigma.

### 3.2 Stigma: Self‐stigma

When considering what life as a carrier would be like, many participants felt it would be very different from their life currently and showed similar stigma as expressed above. It was common for being a carrier to be viewed as some kind of “deficit” (participants' words, not ours). For example, when asked what life as a carrier would be like, one participant said:

I guess it could hard for me to find someone willing to raise children with me if I have a really bad genetic deficit. (P10)

Similarly, other participants hinted at the fact that they would assume potential partners would discriminate against them. This participant suggests they might inherently value themselves less and some participants even stated explicitly that they viewed their hypothetical carrier status as a sign of weakness:

I know some people won't think like that but personally like I would like that's on me. I've given her my genes…and like there is clearly like a weakness in the line do you know what I mean? (P18)

The use of the language “line” to talk about inheritance is an abbreviation of “bloodline” and has historically been used in contexts, particularly in relation to royal families—with connotations of deciding people's worth, and desirability for marriage which would today be seen as discriminatory. This describes an intense feeling of inferiority and inadequacy, caused by the guilt of being a carrier.

### 3.3 Stigma: Creation—schematic knowledge

Judgments of carriers were notably quick among participants and many justified stigmatizing views using quick, unintentional misconceptions. Much of the stigma observed often stemmed from confusion that carriers in some way had the condition. Of note, this was reported in interviews after participants had received information about carriers available via the NHS.

I would probably immediately think that they like had the full condition or illness or whatever. I would think they would show the symptoms of it. (P23)

This idea of carriers as sufferers was pervasive across participants, particularly those who expressed stigmatizing views of carriers. However, some participants did understand that carriers would not have medical effects of the condition. These participants remained confused about exactly what it meant to be a carrier of a genetic condition: “I mean they do have the disease but they don't have the symptoms” (P5). This particular participant went on to refer to genetic condition sufferers as “symptomatic carriers” at other points during the interview. Probing revealed participants were confused by the fact that carriers, who were explained in the diary entries as not having any medical symptoms, could still pass on the condition without actually having it. Many expressed the idea that if people can pass on a condition, then they *must* have it, even if they do not have symptoms, analogous to coronavirus. From this, we could see that there was a jarring between existing knowledge systems (schemas) and this novel information, which was leading to the stigma.

Another observation present across multiple, albeit fewer, interviews was the idea that carrier status may mean that the effects of the condition were currently hidden, but may manifest later in life: “I'm scared if the condition will kick in and how it will affect me drastically in the future” (P12). Some participants, however, could correctly explain the meaning of carrier status and did not express stigmatizing views as frequently:

I think it would just be annoying to know that I am no different and I think that's what would be the issue it's that I don't understand why people have this perception when there is no difference between … it's not going to affect your life, there is no need to be like that really. (P23)

This participant had studied A‐level biology and could confidently explain recessive inheritance. However, these examples of correct genetic knowledge reducing stigma were rare even among a highly educated sample and may be best viewed as “the exception that proves the rule” regarding quick schematic judgments leading to stigma.

### 3.4 Impact on relationships: Familial

Participants were concerned that their decision to have a child would affect their relationship with their immediate family members, with many fearing that family members' views on carriers would be predominantly negative:

Psychologically, I would feel myself as a burden; it just feels really stressful, that I am bringing such a hassle to my family. (P12)

Others suggested their family would question their decision to have a child knowing there was the potential for them to be a carrier:

I don't know if they knew that I knew before I had the child, if they would then be like why did you have the child if you knew that they were going to be a carrier. (P14)

For one participant, it was clear that their close family had such strong negative pre‐conceptions of carriers; they were opposed to them even starting a relationship with a carrier:

I remember my grandad always saying to my mum like we've always had a strong family line like there's never been anything wrong with our side so don't try and get with anyone that kind of has like genetic implications. (P18)

Intertwined with their family's explicit thoughts on carriers in the context of romantic relationships, there was a clear distinction between “them” and “us,” communicated through carrier status being something “wrong” with someone, and something that made them weak, in contrast to this participants “strong” family line. While it was not clear from this participant's interview whether their family had first‐hand experience of a genetic condition, there certainly was a powerful understanding within that family that genetic conditions were to be avoided. However, some participants did state they would be pleased about receiving this information as they were aware of the difference between being a carrier and actually having a genetic condition, and so would be grateful they were just a carrier.

### 3.5 Impact on relationships: Romantic

When participants reflected on how they would feel disclosing their carrier status to a partner, it was common for participants to highlight the reproductive implications of carrier status as a barrier in romantic relationships:

like nervous and anxious that he might act… yeah aversely and might yeah decide not to… want to be with me out of not‐ out of like that life risk for a child and like not wanting that for the child which I would think is fair enough (P13)

The justification of ending a relationship as a young adult due to future reproductive issues was perceived as “fair enough,” highlighting a belief from some participants that carriers are undesirable for romantic relationships.

Even when participants understood that the child would only inherit the condition if both parents were carriers, the perceived risk for the child often resulted in dismissing the option of having biological children:

if it was something that was quite high risk, negative, genetically to be passed down […] I would have to come to like a compromise with them maybe if we was going to have kids, or if I really wanted kids, I'd have to like go a separate way (P24)

Although the possibility of compromise was discussed, this particular participant perceived that if the reproductive risk was too great, they felt they would rather sacrifice the relationship than pursue non‐biological ways of becoming parents. Although this at first may seem to contradict the above narrative about dismissing biological options for having children, it is achieving the same ends by different means—participants are still avoiding the chance of having a child with a condition; they would rather end the relationship than pursue assisted reproductive means. Although not all participants said this, the implication that others perceive carriers as undesirable could be detrimental to carriers' self‐concept.

It is important to note, however, that some believed that knowing their carrier status was positive because it would enable them to make an informed decision with their partner. Such participants believed they could overlook their desire for a biological child, for example:

I feel like I'm quite resilient so I think like if we want to have children together and it's not safe to do so, we'd go to the doctors […] and obviously it is upsetting but as long as I can have some sort of child with my partner then I don't mind (P6)

It was clear that this participant considered being resilient as integral to overcoming the challenges associated with having a child with someone with known carrier status. Having a child with a carrier was viewed as “unsafe” and while medical input from a doctor would be “upsetting,” this was viewed as unavoidable and necessary to have a biological child.

Ultimately, there was a sense that participants feared judgment from both their family members and romantic partners, and were concerned that either may respond negatively to their own or their child's carrier status. For many participants, knowledge of carrier status presented a barrier to having future children and depending on the relationship status, forced participants to consider whether a relationship should end or was even worth starting in the first place.

### 3.6 Impact on relationships: Parent–child relationship

Many participants felt anxious about the possibility of having a child who was a carrier with a strong sense that the participants' own carrier status would have a significant psychological impact on their decision to have a child:

I think that being a carrier would put a lot of pressure on someone, psychologically, the uncertainty of whether to have children based on whether you think it was worth the risk is a huge burden. (P15)

Concerns about their future child's carrier status also appeared to be linked to a fear of judgment from others for having gone through with the pregnancy knowing the potential for their child to be a carrier beforehand:

If my child were a carrier, I would be particularly concerned that I would be blamed. (P9)

Others sought reassurance that their decision to have a child would not cause their child to suffer:

I would feel very guilty that I have caused this; I would want reassurance that my child would be okay and will not suffer because of this. (P17)

Some participants felt that if their child was a carrier, this would not only have a significant psychological impact on them but would also affect their child's life with their child facing the same reproductive decision burden they faced now:

I guess if I have known that I had it beforehand then I'd feel really bad cuz [sic] I'd be like, I kind of knew the risks. I still chose to do that. So, I'd feel like it's kind of I'm partially to blame for my child to have to go through that as well. (P3)

For other participants, the reproductive decision burden was too overwhelming; with one participant saying that choosing to have a child would be cruel:

Would you go ahead with having kids when they could have like a life‐limiting disease? I think that's cruel personally (P18)

This led to some saying they would choose not to have their own biological child at all:

I would probably think about whether to adopt or not. I know that's a bit harsh, but I don't really want a child that I'm going to have to look after in more ways than a normal parent would. I would rather reduce that possibility (P5)

Participants frequently suggested that the implications of a child being a carrier or having a genetic condition were both inherently negative and, crucially, they viewed the implications of both being a carrier and having the condition similarly negatively:

I think it's obviously not something any parent wants for their child and I think it would be hard knowing that I was a component in them developing either carrier status or the condition itself. (P23)

Crucially, this participant does differentiate between being a carrier or having a condition, and yet still highlights a negative impact on parents of passing this on. For many participants, the reproductive decision burden was underpinned by lack of knowledge about the difference between carrier status and condition, leading many to feelings of guilt and self‐blame, and others to choose not to have children at all.

When asked about how participants would respond to being informed of their child's carrier status, the recurrent emotion participants described was guilt: “I think I might feel a bit guilty… cause I was a carrier… so that's why my child is a carrier…” (P21). For some participants this sense of guilt manifested as even more intensely negative emotions: “I would feel very responsible and shameful… I just feel like I've done something really bad” (P17). Another participant even went as far as to question whether having a child who is a carrier would be “a punishment for people who did bad things during previous lives” (P19).

Other participants were worried that the distress they felt about being a carrier would naturally transfer to their carrier child: “The distress that they then might go through in processing that in probably the same way that I have” (P14). Indeed the idea of transference, both genetically in terms of their child inheriting his or her carrier status from them and in terms of the psychological impact transferred to the child, was communicated by many participants: “I’d feel guilty that if the child was a carrier, I’ve just passed on the burden” (P9).

However, not all participants' responses to their child's carrier status were inherently negative, and two participants described how they would feel the opposite: “I'd be quite relieved that they haven't actually got it and I could help them if they had any issues with it” (P2) with others “So grateful that they don't have the condition” (P16). Importantly, participants who held previous formal education expressed relief and gratitude that their child was a carrier as opposed to suffering the condition, whereas participants who claimed little or no prior genetic knowledge often expressed guilt and shame.

### 3.7 Communication challenges: Timing

When asked when carrier status should be disclosed within a romantic relationship, a number of participants made it clear that they would prefer disclosure to occur early on in a relationship and that carriers have a certain responsibility to do so:

if they disclose that later I wouldn't blame them but… yeah it would be nicer to know early on and then just have that in the back‐ well in the back of your mind sort of because it's actually quite a deep thing (P13)

This preference for early disclosure reflects the extra considerations that may be taken when entering a relationship with a carrier. Additionally, the mention that this participant would keep knowledge of their partners' carrier status in the “*back of [their] mind*” highlights how some participants viewed carrier status as something that would not be dismissed as trivial.

Despite commonly acknowledging the difficulty of disclosure, other participants felt that if their partner was a carrier, they themselves were entitled to know, relating this to trust and honesty within the relationship:

Umm I think the most important thing is there shouldn't be deception like yeah if my romantic partner is a carrier then umm he should tell me this. Umm I would give support but umm I think I have the right to know yeah (P10)

The use of the word “deception” is interesting. When we put this into the context of what else people feel they would have a right to know about their partner, it indicates the importance people place on this information. Although this sense of the requirement for disclosure was predominant, a few participants spoke of disclosure in a more casual way:

I think I would tell someone within like a month or something maybe [INT: Yeah] yeah it's not a case of that we're having kids, I think for me it would more of a ‘just so you know.’ (P6)

Here, their preference for early disclosure reflected their perception that carrier status was not a big deal so they would not fear an adverse reaction. While only a few participants responded this way, across participants this response occurred where it was believed both partners would understand that carrier status would only pose reproductive issues if both individuals were carriers.

While some felt knowledge of their partners' carrier status was most important in the context of reproductive decision‐making, they also felt that disclosure should be more casual, highlighting their understanding of recessive carrier inheritance:

I don't know I think it should definitely be before you talk about having children. But I don't think it needs to be a big announcement I don't think you need to like write it in the sky or anything (P25)

Ultimately, despite carrier status disclosure being perceived as an anxiety‐provoking experience, many participants believed that disclosure should occur early on, and felt that they had a clear right to know their partners' health information. The few who spoke more casually about disclosure had previously discussed the insignificance of carrier status unless both individuals were carriers, and others who saw no need for disclosure at all had no desire for children.

### 3.8 Communication challenges: Projected reactions

When asked to reflect on how they would feel disclosing their carrier status to a potential partner, most believed they would feel anxious due to fear of judgment. Disclosure was frequently perceived as an unnerving experience with carrier status inherently viewed as a flaw, potentially increasing the likelihood of a relationship ending:

I would be worried if they were just going to up and leave [INT: mhmm] just like straight away, worried that their opinion of me possibly could change for I don't know maybe they had a reason that they didn't want to be with someone who was a carrier. (P24)

For context, this participant held these rejection concerns even though their only concern about their partner being a carrier was related to future reproduction. This was a common pattern across the interviews and this fear of judgment from others appeared routed in concerns that others would misunderstand carrier status. These data also show that there could be difficulties in balancing what is important for others to know against your own interests of not jeopardizing the relationship early.

However, although some participants believed they would feel anxious for fear of rejection after their disclosure of their own carrier status, others discussed a lack of judgment if their partner disclosed their carrier status:

I wouldn't be judgmental about it. I'd actually respect them more for telling me if they did because that's quite a personal thing to share and the fact that they've trusted me with that information I'd feel quite respectful. (P11)

The admiration this participant believes they would have after disclosure highlights some participants' perceptions that disclosure would increase relational closeness. This contrasted with the common expectation that others would be stigmatized.

When asked to reflect on how they would disclose carrier status to a partner, one participant discussed the need for intoxication:

I'd probably tell them when I was drunk like that's the only way I can see myself, like I don't think I'd be able to bring it up sober I'd just like bring it up on a night out. (P20)

This participant also reported that if their partner disclosed they were a carrier, this would cause them to consider whether a relationship with someone else would be better.

Many participants also thought that the link of the term carrier with COVID‐19 would cause issues:

if people haven't got an awareness of what a carrier of say, sickle cell could be, but they know that a carrier of Covid and then associated that with negative because it's Covid, and you're scared and like you say it's a health hazard then people will view a carrier of sickle cell or thalassemia as a negative health hazard when… it's actually not. (P6)

Disclosure was perceived to be more difficult for carriers if others generalized the negative connotations of COVID‐19 to genetic carriers. One participant even advised that carriers keep their experiences private while the population was so exposed to COVID‐19 coverage:

so yeah it's just a sensitive topic that's likely to have a negative idea related to it so talking about it would be would probably be not the best idea at the moment. (P21)

This account illustrates many participants' perception that carriers' experience of disclosure would be increasingly challenging during the COVID‐19 pandemic.

## 4 DISCUSSION

When trying to understand the impact of carrier identification on individuals via NBS, emerging adulthood, is a key age to explore. At this stage, the reaction of peers and the formation of romantic relationships are central elements of identity formation. This is the first study to explore the views of young adults about carrier status after the COVID‐19 pandemic. Most participants had little knowledge about carriers and so used their schematic (Hess & Slaughter, 1990) or pre‐existing knowledge of conditions when considering the impact of carrier status. This gave a false sense of certainty about knowledge as also seen in Noke and Ulph (2014) where they are sure the information is correct, but can be seen using it in the wrong context. A systematic review of parents' reactions to NBS results suggests that this could be due to people's cognitive models of illness models which view results as binary—either you have a label, symptoms, treatment, etc. or you do not (Johnson et al., 2022). This could explain some of our findings and those who have previously found that people believed autosomal carriers would suffer health effects (Mayo‐Gamble et al., 2018) or that a child was more likely to be a carrier if the mother was older (McClaren et al., 2008). What is notable in our study, however, was that people persisted in doing this even after engaging in the diary study where they were asked to engage with information about carriers produced by the NHS and reflect on it.

Unfortunately, our data suggest that this way of understanding carrier status is leading to stigma. The finding was consistent with previous research showing that people stigmatize carriers (e.g., Evers‐Kiebooms et al., 1994) and that people are concerned about informing their family and their family's judgment (Ulph et al., 2011). Although our sample could be seen as a “naïve sample” (they were not carriers and had no reason to be engaging with literature on genetic carriers), one interviewee could recount a family member warning against relationships with carriers. This fits with other research that has found messages that carriers may be discriminated against in terms of choosing a life partner (Locock & Kai, 2008; Ulph et al., 2011). Within our sample, participants were even using predicted views/reactions of others (as they had not in reality discussed this) to form their own reproductive decisions and that they were aware of and sensitive to the judgments of others.

The belief that carrier status could be punishment for an individual's reactions is worth highlighting even though it only occurred once. The Piagetian theory of illness understandig (Bibace & Walsh, 1980), one of the theories that seeks to explain how illness beliefs develop, would view this thinking as “immanent justice”, this style of thinking is attributed to younger children. However, this belief was found in our group in a scientifically literate group of young adults. This would fit with functionalist theories of illness understanding, which suggests that people can have a varied profile of illness knowledge based on their experiences and exposure. Although we hope these beliefs are rare, it does highlight that even scientifically literate adults can have harmful beliefs about topics over which they have little knowledge. Our findings suggest such stigma, or concerns about being stigmatized, may have been exacerbated by the COVID‐19 pandemic narrative. This awareness of judgment led many interviewees to state that there would be anxiety surrounding disclosure of their carrier status to peers, especially in romantic relationships.

The use of a qualitative design in this study is a strength as there are concerns about the use of quantitative measures to explore impact of genomic labels (Biesecker et al., 2013). We tried to limit the influence of disease knowledge by simply using the term “carrier” rather than linking it to a specific condition—thus making the findings more transferable. The use of diaries was critical in enabling our participants to engage with and consider the research topic in‐depth (Alaszewski, 2006) before taking part in the interview. Without this, interviews run the risk of collecting responses that are more likely to parrot socially acceptable views, without really considering what reactions would be. As mentioned in the procedure, prior to every interview, the diaries were summarized, and each interview incorporated the key messages. Therefore, we are confident that this paper does not miss any of the central messages contained in that data. Our interviews were conducted online. Research comparing face‐to‐face interviews with those conducted online using video‐conferencing software found not only that there was no difference in interview quality (Cabaroglu et al., 2010) but also that participants were more open and expressive (Deakin & Wakefield, 2013) which was our experience. Through the screen share function, we were able to further about participants' diary responses and gave participants the space to expand on what they had written, positively contributing to the breadth and depth of data. Although we believe the use of TA to analyze the data has been beneficial given the aims of this study and intended audience, we found individuals hold conflicting views on this topic. To understand those conflicts and their implications an interpretative phenomenological study (Smith et al., 2021), a form of in‐depth research that can take a case study approach, enabling result narratives to give a sense of the individual and how their views track across the findings would be valuable. Caution must be taken as however well‐considered the responses, these are hypothetical situations. However, they do provide insight into the underlying beliefs and where it could be useful to provide information to reduce stigmatization or distress. Likewise, caution should be taken, as the sample is largely female, well educated, and white. Similarly to Noke and Ulph (2014), our sample had low levels of genetic knowledge and they were not confident in their knowledge. Some mentioned knowing people with a genetic condition and further research exploring how this impacts views and understanding would be warranted. Yet, as with the Noke and Ulph paper, we again found that even in this well‐educated sample, there were indicators to suggest the merits of an improved general awareness and understanding of what it truly means to be an autosomal carrier.

The findings of this study show that young adults engaging in romantic relationships expect their partner to disclose this information. However, the findings also add that carriers could be stigmatized, particularly in romantic relationships and more so where there are definite reproductive implications. Although further research should explore this in more diverse samples, the finding warrants further careful consideration about the relative benefits and harms of identifying autosomal carriers before young adulthood if it impacts their romantic relationship formation. They also highlight an urgent need to ensure that we do not assume that knowledgeable adults will not hold harmful beliefs if we provide them with carrier information (as was done in this study). Rather it suggests there is a need to work more broadly to ensure that populations are helped to adjust some of the cognitive models they have on how and why people get a medical label, to help people understand the implications of autosomal recessive carrier status for themselves and others.

### 4.1 Practice implications

Genetic counselors are well placed to shape initiatives to help people understand the implications of being an autosomal recessive carrier to reduce stigmatizing behaviors. They have direct access to families where such discussions are involved but should also be a key voice in public health policies. The consideration of how genomic information impacts social development is especially urgent as screening programs are trialing whole‐genome sequencing, which will increase the number of young adults in receipt of genomic labels.

## AUTHOR CONTRIBUTIONS

Fiona Ulph confirms that they had full access to all the data in the study and take responsibility for the integrity of the data and the accuracy of the data analysis. All of the authors gave final approval of this version to be published and agreed to be accountable for all aspects of the work in ensuring that questions related to the accuracy or integrity of any part of the work are appropriately investigated and resolved.

## CONFLICT OF INTEREST STATEMENT

All authors declare that they have no conflict of interest.

## ETHICS STATEMENT

Human studies and informed consent: Approval to conduct this human subjects research was obtained by the University of Manchester Research Ethics Committee. All procedures followed were in accordance with the ethical standards of the responsible committee on human experimentation (institutional and national) and with the Helsinki Declaration of 1975, as revised in 2000. Informed consent was obtained from all patients to be included in the study.

## Supporting information


Appendix S1

## Data Availability

Data from this study are not available to share due to remit of informed consent given.
