# Supplementary material for: Understanding the psychological impact of identifying carrier status on young adults: A qualitative study exploring peer reactions
Source: J Genet Couns. 2024 Apr 26;34(1):e1903. doi: 10.1002/jgc4.1903 (PMC11735185; doi:10.1002/jgc4.1903)
Supplement: Supplementary file 1 — Appendix S1 [file JGC4-34-0-s001.docx]

DIARY

DAY ONE

**By returning this document you are indicating you consent to the data being included in our study.**

Today we just want to start you off thinking about what it means to be a carrier of a condition. We’d like to explore the beliefs and knowledge you already have, even if the honest answer is you don’t know much! So we’re asking you to think of the word carrier and then answer the following questions:

- What would you assume it is?
- How serious do you see it being?
- What do you know of the treatments?
- How do you think it affects someone socially or psychologically?
- What would be your first questions about it?

___________________________________________________________________________

___________________________________________________________________________

___________________________________________________________________________

___________________________________________________________________________

___________________________________________________________________________

___________________________________________________________________________

___________________________________________________________________________

___________________________________________________________________________

___________________________________________________________________________

Exercise

Now we would like you to note down any characteristics that come to mind for an individual who is a carrier and an individual who is a non-carrier (there is no right or wrong answer).


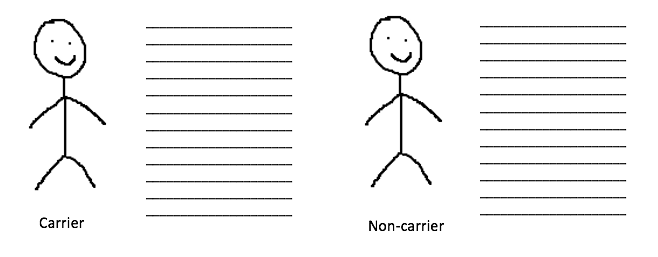


________________________________________________________________________________________________________________________________________________

________________________________________________________________________________________________________________________________________________

DAY TWO

A carrier is someone who has inherited a genetic (or autosomal recessive) condition. Importantly, being a carrier of an autosomal recessive condition does **not** cause medical problems *(NHS, 2015)*. However, if you have a child with someone who is also a carrier, there is a 1 in 4 chance that your child could have the condition.

Today we’ll look at the self and internalisation (how finding out you were a carrier would affect your views of yourself). We want to start with a “thought experiment” where we want you to imagine you have just been told you are a carrier of a genetic condition:

- Having just been told that you are a carrier of a genetic condition, what are the first questions that spring to mind that you need answers to?
- How do you feel?
- What support, if any, do you think you’d need?
- How do you think others will view you?
- Anything else you are thinking or feeling right now linked to this.

___________________________________________________________________________

___________________________________________________________________________

___________________________________________________________________________

___________________________________________________________________________

___________________________________________________________________________

___________________________________________________________________________

___________________________________________________________________________

___________________________________________________________________________

___________________________________________________________________________

- Having read the carrier definition above, coupled with the knowledge that you yourself are a carrier, we would like you to complete the same task as yesterday and note down any characteristics that come to mind for an individual who is a carrier and an individual who is a non-carrier (there is no right or wrong answer) and then answer the questions below.


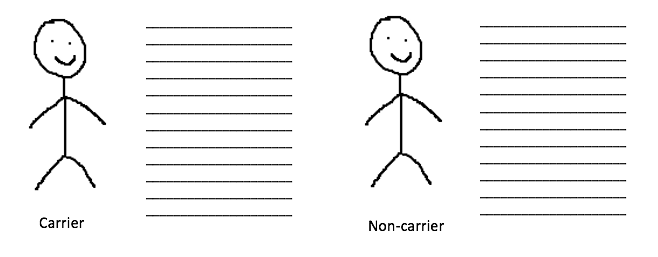


________________________________________________________________________________________________________________________________________________

________________________________________________________________________________________________________________________________________________

Throughout the day, be aware of what you hear and think related to this topic and note down here all the connections you have made.

(We want this to be like a normal diary entry, capturing your REAL experiences in this day in relation to the IMAGINED scenario that you are a carrier.)

- What did you hear? Read? See? That is linked to the imagined scenario that you are a carrier.
- Did you look for any information? If so, where?

___________________________________________________________________________

___________________________________________________________________________

___________________________________________________________________________

___________________________________________________________________________

___________________________________________________________________________

___________________________________________________________________________

___________________________________________________________________________

___________________________________________________________________________

___________________________________________________________________________

Lastly, we would like to know if your response to the previous question has been influenced by anything that you have been exposed to about the Covid-19 pandemic today? (e.g. something you heard, or read?)

___________________________________________________________________________

___________________________________________________________________________

___________________________________________________________________________

___________________________________________________________________________

___________________________________________________________________________

___________________________________________________________________________

___________________________________________________________________________

______________________________________________________________________________________________________________________________________________________

DAY THREE

Yesterday, we did a thought experiment where you considered what it would be like to be told you were a carrier of a genetic condition.

Today, we would like you to complete an exercise that explores who you might tell if you were told you are a carrier and how close these people are to you.

In life, it’s common to know a wide range of people. Some are very close to you, known as the inner circle (e.g. your best friend, some family members). Others are people in our lives that we share no personal information with, known as the outer circle. These tend to be people like some course mates, friends of friends, distant family members. Lastly, there is a middle circle where they may tell people things in some circumstances (sports coach and physical injury), but not in others (telling your sports coach about the breakdown of a romantic relationship).

Can you list people in your different circles – these do not need to be their real names, they are just so you know who you’re referring to [i.e. mum, aunt x, coach y] then let us know whether you think you would have told them by now.

________________________________________________________________________________________________


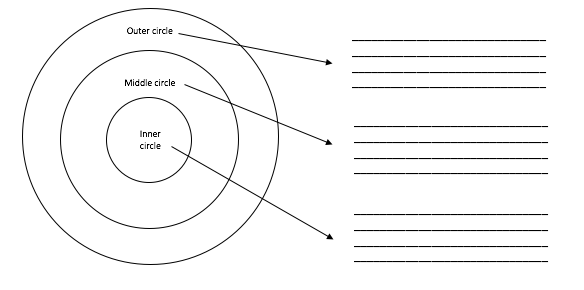


________________________________________________________________________________________________

________________________________________________________________________________________________

Your thoughts about yourself

- Do you have any new questions now you’ve had time to think?
- How do you feel?
- What support, if any, do you think you’d need?
- How do you think others will view you?
- Anything else you are thinking or feeling right now linked to this.

___________________________________________________________________________

___________________________________________________________________________

______________________________________________________________________________________________________________________________________________________

Can you complete your thoughts about characteristics of carriers and non-carriers?


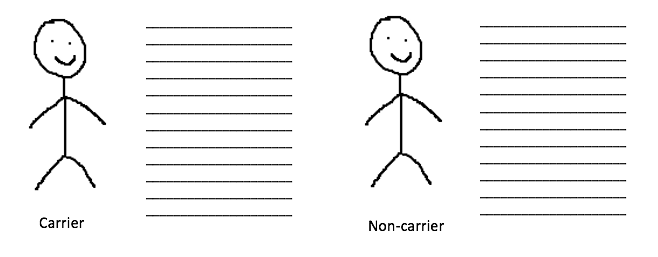


________________________________________________________________________________________________________________________________________________

________________________________________________________________________________________________________________________________________________

Throughout the day, be aware of what you hear and think related to this topic and note down here all the connections you have made.

(We want this to be like a normal diary entry, capturing your REAL experiences in this day in relation to the IMAGINED scenario where you have just been told that you are a carrier.)

- What did you hear? Read? See? That is linked to the imagined scenario that you are a carrier.
- Did you look for any information? If so, where?

___________________________________________________________________________

___________________________________________________________________________

___________________________________________________________________________

___________________________________________________________________________

___________________________________________________________________________

___________________________________________________________________________

___________________________________________________________________________

______________________________________________________________________________________________________________________________________________________

Lastly, we would like to know if your response to the previous question has been influenced by anything that you have been exposed to about the Covid-19 pandemic today? (e.g. something you heard, or read?)

___________________________________________________________________________

___________________________________________________________________________

___________________________________________________________________________

___________________________________________________________________________

___________________________________________________________________________

___________________________________________________________________________

___________________________________________________________________________

______________________________________________________________________________________________________________________________________________________

DAY FOUR

Today, if you haven’t already, we’d like you to think forward to the scenario where you are becoming a parent. Imagine that it is a few years on and you have just given birth to a child who had newborn screening for genetic conditions. You have been told that your child is also a carrier of a genetic condition. Please document your initial thoughts below:

___________________________________________________________________________

___________________________________________________________________________

___________________________________________________________________________

___________________________________________________________________________

___________________________________________________________________________

___________________________________________________________________________

___________________________________________________________________________

______________________________________________________________________________________________________________________________________________________

Now you’ve known that you’re a carrier for 3 days, has who you have told changed? Update this diagram to show who you think you would have told now:


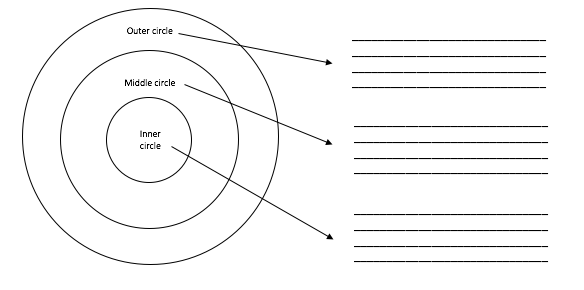


________________________________________________________________________________________________

________________________________________________________________________________________________

Your thoughts about yourself

- Do you have any new questions now you’ve had time to think?
- How do you feel?
- What support, if any, do you think you’d need?
- How do you think others will view you?
- Anything else you are thinking or feeling right now linked to this.

___________________________________________________________________________

___________________________________________________________________________

______________________________________________________________________________________________________________________________________________________

___________________________________________________________________________

___________________________________________________________________________

______________________________________________________________________________________________________________________________________________________

Can you complete your thoughts about characteristics of carriers and non-carriers?


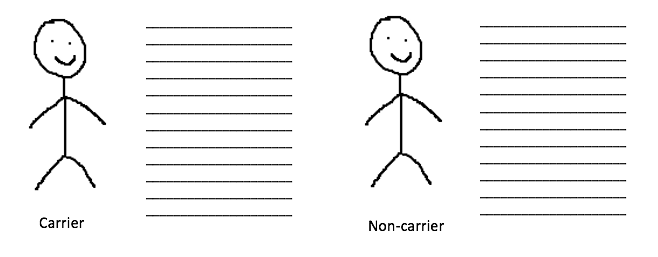


________________________________________________________________________________________________________________________________________________

________________________________________________________________________________________________________________________________________________

Throughout the day, be aware of what you hear and think related to this topic and note down here all the connections you have made.

(We want this to be like a normal diary entry, capturing your REAL experiences in this day in relation to the IMAGINED scenario where you have just been told that you are a carrier.)

- What did you hear? Read? See? That is linked to the imagined scenario that you are a carrier.
- Did you look for any information? If so, where?

___________________________________________________________________________

___________________________________________________________________________

___________________________________________________________________________

___________________________________________________________________________

___________________________________________________________________________

___________________________________________________________________________

___________________________________________________________________________

Lastly, we would like to know if your response to the previous question has been influenced by anything that you have been exposed to about the Covid-19 pandemic today? (e.g. something you heard, or read?)

___________________________________________________________________________

___________________________________________________________________________

___________________________________________________________________________

___________________________________________________________________________

___________________________________________________________________________

DAY FIVE

You have now known that you are a carrier for 4 days. Please document below your thoughts about yourself:

- Do you have any new questions now you’ve had time to think?
- How do you feel?
- What support, if any, do you think you’d need?
- How do you think others will view you?
- Any further thoughts about how you might feel about being told your newborn baby is a carrier?
- Anything else you are thinking or feeling right now linked to this.

___________________________________________________________________________

___________________________________________________________________________

______________________________________________________________________________________________________________________________________________________

___________________________________________________________________________

___________________________________________________________________________

______________________________________________________________________________________________________________________________________________________

Now you’ve known that you’re a carrier for 4 days, has who you have told changed? Update this diagram to show who you think you would have told now:


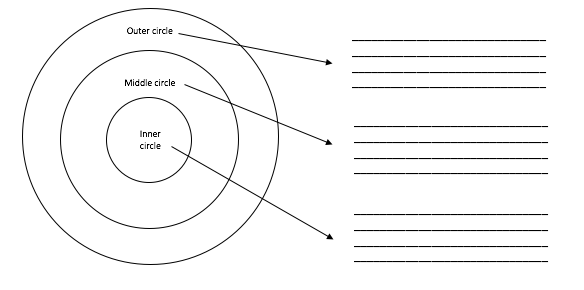


________________________________________________________________________________________________

________________________________________________________________________________________________

________________________________________________________________________________________________

Can you complete your thoughts about characteristics of carriers and non-carriers?


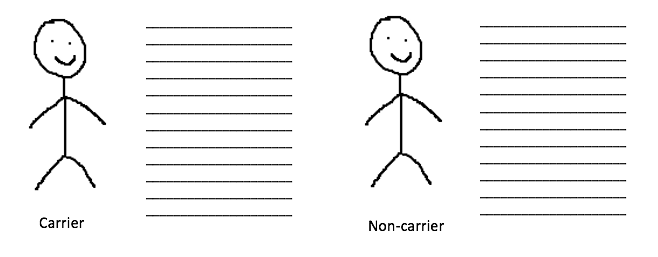


________________________________________________________________________________________________________________________________________________

Throughout the day, be aware of what you hear and think related to this topic and note down here all the connections you have made.

(We want this to be like a normal diary entry, capturing your REAL experiences in this day in relation to the IMAGINED scenario where you have just been told that you are a carrier.)

- What did you hear? Read? See? That is linked to the imagined scenario that you are a carrier.
- Did you look for any information? If so, where?

___________________________________________________________________________

___________________________________________________________________________

___________________________________________________________________________

___________________________________________________________________________

___________________________________________________________________________

___________________________________________________________________________

___________________________________________________________________________

______________________________________________________________________________________________________________________________________________________

Lastly, we would like to know if your response to the previous question has been influenced by anything that you have been exposed to about the Covid-19 pandemic today? (e.g. something you heard, or read?)

___________________________________________________________________________

___________________________________________________________________________

___________________________________________________________________________

___________________________________________________________________________

___________________________________________________________________________

___________________________________________________________________________

___________________________________________________________________________

______________________________________________________________________________________________________________________________________________________

DAY SIX

You have now known that you are a carrier for 5 days. Please document below your thoughts about yourself:

- Do you have any new questions now you’ve had time to think?
- How do you feel?
- What support, if any, do you think you’d need?
- How do you think others will view you?
- Any further thoughts about how you might feel about being told your newborn baby is a carrier?
- Anything else you are thinking or feeling right now linked to this.

___________________________________________________________________________

___________________________________________________________________________

______________________________________________________________________________________________________________________________________________________

___________________________________________________________________________

___________________________________________________________________________

______________________________________________________________________________________________________________________________________________________

Now you’ve known that you’re a carrier for 5 days, has who you have told changed? Update this diagram to show who you think you would have told now:


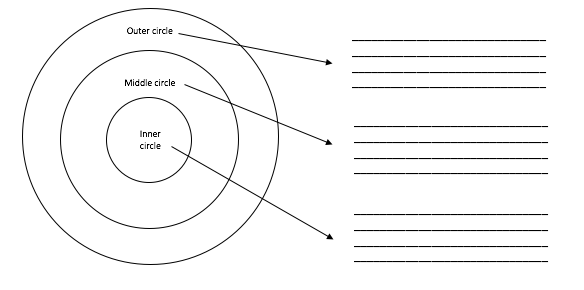


________________________________________________________________________________________________

________________________________________________________________________________________________

________________________________________________________________________________________________

Can you complete your thoughts about characteristics of carriers and non-carriers?


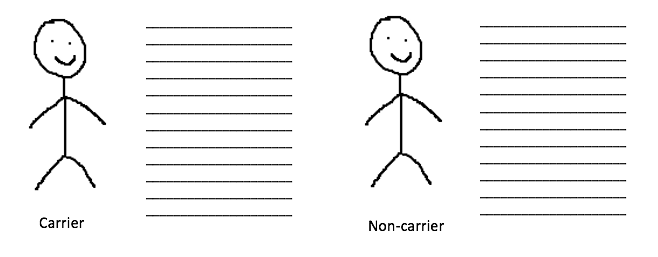


________________________________________________________________________________________________________________________________________________

________________________________________________________________________________________________________________________________________________

________________________________________________________________________________________________________________________________________________

________________________________________________________________________________________________________________________________________________

Throughout the day, be aware of what you hear and think related to this topic and note down here all the connections you have made.

(We want this to be like a normal diary entry, capturing your REAL experiences in this day in relation to the IMAGINED scenario where you have just been told that you are a carrier.)

- What did you hear? Read? See? That is linked to the imagined scenario that you are a carrier.
- Did you look for any information? If so, where?

___________________________________________________________________________

___________________________________________________________________________

___________________________________________________________________________

___________________________________________________________________________

___________________________________________________________________________

___________________________________________________________________________

___________________________________________________________________________

______________________________________________________________________________________________________________________________________________________

Lastly, we would like to know if your response to the previous question has been influenced by anything that you have been exposed to about the Covid-19 pandemic today? (e.g. something you heard, or read?)

___________________________________________________________________________

___________________________________________________________________________

___________________________________________________________________________

___________________________________________________________________________

___________________________________________________________________________

___________________________________________________________________________

___________________________________________________________________________

______________________________________________________________________________________________________________________________________________________

DAY SEVEN

You have now known that you are a carrier for 6 days. Please document below your thoughts about yourself:

- Do you have any new questions now you’ve had time to think?
- How do you feel?
- What support, if any, do you think you’d need?
- How do you think others will view you?
- Any further thoughts about how you might feel about being told your newborn baby is a carrier?
- Anything else you are thinking or feeling right now linked to this.

___________________________________________________________________________

___________________________________________________________________________

______________________________________________________________________________________________________________________________________________________

___________________________________________________________________________

___________________________________________________________________________

______________________________________________________________________________________________________________________________________________________

Now you’ve known that you’re a carrier for 6 days, has who you have told changed? Update this diagram to show who you think you would have told now:


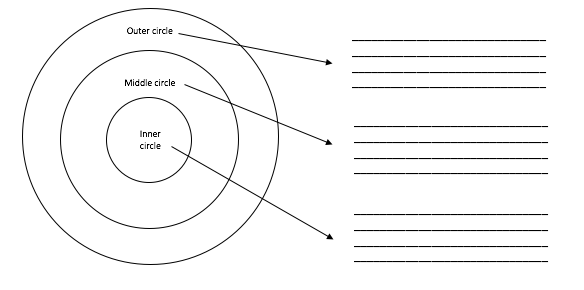


________________________________________________________________________________________________

________________________________________________________________________________________________

________________________________________________________________________________________________

Can you complete your thoughts about characteristics of carriers and non-carriers?


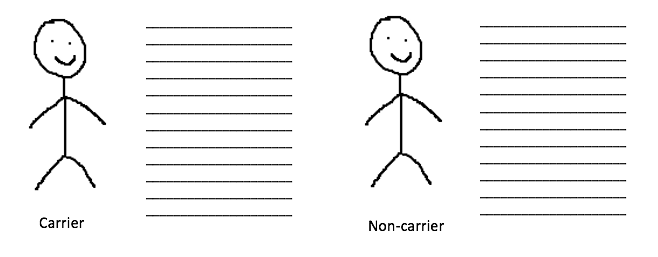


________________________________________________________________________________________________________________________________________________

________________________________________________________________________________________________________________________________________________

Throughout the day, be aware of what you hear and think related to this topic and note down here all the connections you have made.

(We want this to be like a normal diary entry, capturing your REAL experiences in this day in relation to the IMAGINED scenario where you have just been told that you are a carrier.)

- What did you hear? Read? See? That is linked to the imagined scenario that you are a carrier.
- Did you look for any information? If so, where?

___________________________________________________________________________

___________________________________________________________________________

___________________________________________________________________________

___________________________________________________________________________

___________________________________________________________________________

___________________________________________________________________________

___________________________________________________________________________

______________________________________________________________________________________________________________________________________________________

Lastly, we would like to know if your response to the previous question has been influenced by anything that you have been exposed to about the Covid-19 pandemic today? (e.g. something you heard, or read?)

___________________________________________________________________________

___________________________________________________________________________

___________________________________________________________________________

___________________________________________________________________________

___________________________________________________________________________

___________________________________________________________________________

___________________________________________________________________________

______________________________________________________________________________________________________________________________________________________
